# Heavy-Atom Tunneling in the Covalent/Dative Bond Complexation of Cyclo[18]carbon–Piperidine

**DOI:** 10.1021/acs.jpcb.2c00218

**Published:** 2022-02-18

**Authors:** Ashim Nandi, Jan M. L. Martin

**Affiliations:** Department of Molecular Chemistry and Materials Science, Weizmann Institute of Science, 7610001 Reḥovot, Israel

## Abstract

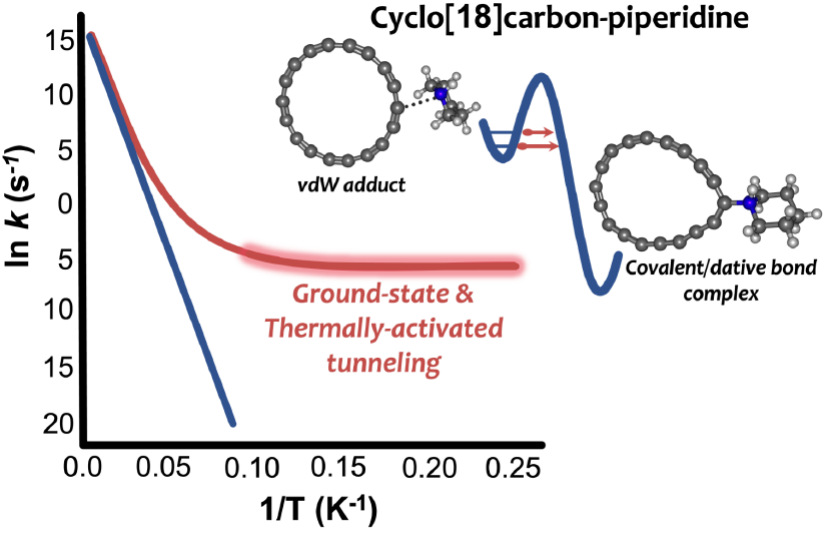

Recent quantum chemical computations demonstrated the electron-acceptance behavior of this highly reactive cyclo[18]carbon (C_18_) ring with piperidine (pip). The C_18_–pip complexation exhibited a double-well potential along the N–C reaction coordinate, forming a van der Waals (vdW) adduct and a more stable, strong covalent/dative bond (DB) complex by overcoming a low activation barrier. By means of direct dynamical computations using canonical variational transition state theory (CVT), including the small-curvature tunneling (SCT), we show the conspicuous role of heavy atom quantum mechanical tunneling (QMT) in the transformation of vdW to DB complex in the solvent phase near absolute zero. Below 50 K, the reaction is entirely driven by QMT, while at 30 K, the QMT rate is too rapid (*k*_T_ ∼ 0.02 s^–1^), corresponding to a half-life time of 38 s, indicating that the vdW adduct will have a fleeting existence. We also explored the QMT rates of other cyclo[*n*]carbon–pip systems. This study sheds light on the decisive role of QMT in the covalent/DB formation of the C_18_–pip complex at cryogenic temperatures.

## Introduction

Because of its unique electronic and structural features, the newly synthesized sp-hybridized cyclo[18]carbon (C_18_) ring has sparked widespread attention to both theoreticians and experimentalists since its first experimental observation in condensed media in 2019.^1^ The successful *in situ* generation and characterization of this decades-old elusive C_18_ ring via atom manipulation by an atomic force microscope (AFM) tip was a landmark study because of the potential to be an alternate candidate for pure carbon allotropes.^2−4^ Following this experimental feat, several theoretical studies have explored the geometrical structure and stability of the C_18_ ring in the gas phase,^5−10^ and more importantly, a series of interesting properties have been highlighted, such as the electronic and transport properties,^8,11−15^ double aromatic character,^16−18^ dynamics behavior,^6^ and so on—most of these studies revolved around the noncovalent interaction of C_18_ with other elements or molecular entities.

In a recent combined experimental and theoretical study by Hobza’s group,^19^ it was shown that an sp^2^-hybridized carbon allotrope, namely C_60_, forms a strong N → C dative bond with piperidine (pip), thereby explaining the long-standing question on the enhanced stability of C_60_ with pip compared to other organic or inorganic solvents.^20,21^ In contrast, planar carbon allotropes (graphene and nanotubes) form only noncovalent interactions with pip.^22^ Stimulated by these works, the same group theoretically studied the electron-acceptance potential of C_18_ with pip in the gas phase and continuum solvent (using the pip dielectric constant).^23^ According to their DFT computations at the ωB97XD/def2-TZVPP level, the C_18_–pip complex was predicted to exhibit double-well potentials along the N–C reaction coordinate, first forming a weak van der Waals (vdW) adduct with a long N–C bond (3.006 Å), followed by a thermodynamically stable strong dative bond (DB) complex with a short N–C bond (1.501 Å). The vdW → DB transformation was low (activation barrier Δ*E*^‡^ = 3.2 kcal mol^–1^) and highly exergonic (reaction energy Δ*E*_r_ = −12.6 kcal mol^–1^) in the gas phase, taking the vdW complex as a reference. Extra stabilization of the C_18_–pip complex was predicted upon moving to the solvent phase. NBO analysis and molecular dynamics simulation further revealed the stability of the DB complex at room temperature.

Additionally, they have investigated the stability of other hypothetical cyclo[*n*]carbon systems with pip. Noteworthy, prior to the vdW complexation, their computations also predicted a stable hydrogen-bonded complex where hydrogen from N–H forms a bond with C_18_ and its relative energy lies 1 kcal mol^–1^ above the vdW complex in the gas phase. Encouraged by the low activation barrier for the vdW → DB process in C_18_–pip and more importantly by the high exergonicity of the reaction, which may yield a narrow effective barrier width in accord with Hammond’s postulate,^24^ we wondered if this reaction can be driven by heavy-atom quantum mechanical tunneling (QMT) even close to absolute zero.

Reactions involving QMT by heavy atoms (mostly second-row elements of the periodic table)^25−28^ are relatively rare compared to light hydrogen tunneling-based reactions; however, recent years have witnessed the slow emergence of heavy-atom QMT, especially in organic chemistry.^25,28,29^ This nonclassical QMT phenomenon is a well-known effect that significantly accelerates reaction rates by passing through the potential barrier instead of crossing over it.^30,31^ The extent to which QMT occurs in a chemical reaction can be approximated according to , where *P* is the tunneling probability, *w* is the barrier width, Δ*E*^‡^ is the activation energy, and *m* is the mass of the moving parts of the molecule.^26,27^ Clearly, among these three factors that determine the tunneling probability, the barrier width has the most decisive influence on the tunneling probability. Indeed, several experimental and/or computational studies have demonstrated that the characteristic features of reactions driven by heavy-atom QMT are their low and narrow barriers.^25,28,29^ The few documented cases include pericyclic^32^ and degenerate rearrangement reactions involving carbon,^33−35^ fluoride,^36,37^ and boron^38^ tunneling. Carbon and nitrogen tunneling in highly exergonic reactions in reactive intermediate species, such as carbene or nitrene, has also been reported.^39−41^ Recently, boron atom tunneling has been predicted in the highly exergonic reaction involving the N–B bond-stretch isomerization of nitrile–boron halides, whereby the metastable vdW adduct isomerizes to a global minimum covalent/dative bond complex.^42^

In this work, through direct dynamics computations, we show the dominant role of heavy-atom tunneling in the transformation of vdW → DB in the C_18_–pip complex near absolute zero in the solvent phase. We also extend our tunneling results to other cyclo[*n*]carbon–pip (*n* = 14, 16, 18, 20, and 22). This study may elucidate the possibility of leveraging the role of QMT in the dative/covalent functionalization of the C_18_–pip complex.

## Computational Methods

All of the DFT electronic structure calculations were performed at the M06-2X^43^/def2-TZVP level with the Gaussian 16 program suite.^44^ This level of theory was chosen since it correctly reproduces the observed polyynic structure of cyclo[18]carbon.^6,8^ In addition, it was shown to closely match the minimum potential energy surface (PES) of C_18_ against DLPNO-CCSD(T)-F12 computations,^6^ which is crucial for our accurate direct dynamical studies. The SCF convergence criteria were set by using the keyword “opt = tight”, which sets the maximum and root-mean-square (RMS) forces to 1.5 × 10^–5^ and 1.0 × 10^–5^ hartree/bohr and the maximum and RMS displacements to 6.0 × 10^–5^ and 4.0 × 10^–5^ bohr. The “ultrafine” grid, which is a pruned direct product of a 99-point Euler–MacLaurin radial grid combined with a 590-point Lebedev angular grid,^45^ was employed for all DFT calculations. Intrinsic reaction coordinate (IRC) calculations were conducted to confirm that the transition state is smoothly connected to the reactant and product side.

The semiclassical rate constants were computed by using canonical variational transition state theory (CVT),^46^ while the tunneling rates were accounted for by using the highly demanding multidimensional small curvature tunneling (SCT) method.^47,48^ We refer to the semiclassical CVT and the tunneling-corrected SCT rates as *k*_SC_ and *k*_T_. Polyrate17^49^ was used to compute all the rate constants, with Gaussrate17^50^ serving as the interface between Polyrate and Gaussian. The Page–McIver algorithm^51^ with a gradient and a Hessian step size of 0.002 and 0.018 bohr (the smallest standard recommended step size for Polyrate calculation)^49^ was employed to map the reaction energy path for all the studied reactions. Quantized reactant state tunneling (QRST)^52^ calculations were used to obtain accurate rate constants at the subcryogenic temperatures. Geometry optimization and the rate constant calculations in the solvent phase were taken into account by using the integral equation-formalism polarizable continuum model (IEFPCM)^53^ with a dielectric constant of 5.9 (a value for piperidine solvent). For the sake of reproducibility of our QMT computations, and due to the number of keywords involved that can slightly affect the rates, a sample Polyrate input file is provided in the Supporting Information.

All of the energetics, namely binding (BE), threshold barrier (Δ*E*^‡^), and the reaction (Δ*E*_r_) energies, reported throughout this work are in the continuum piperidine solvent phase unless otherwise mentioned and include the zero-point energy corrections.

## Results and Discussion

**Figure 1:**
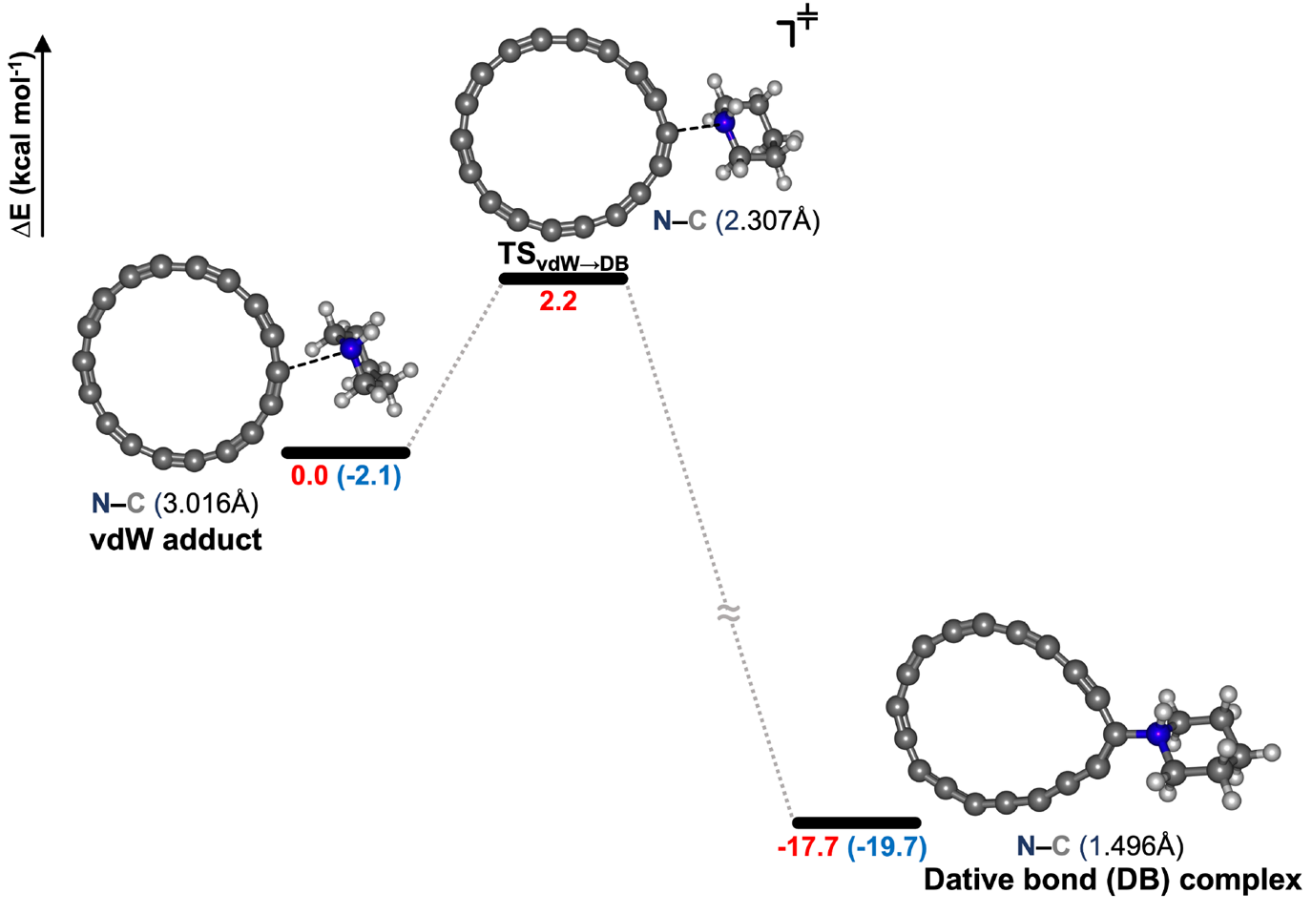
Double-well potential of C_18_–pip complex in the piperidine solvent along with N–C bond distances. The relative energies are indicated in red, and the binding energies within parentheses are in blue.

As a starting point, we considered the double-well potential of the donor–acceptor C_18_–pip complex reported by Hobza and co-workers described above. Our gas-phase computations at the M06-2X/def2-TZVP level yield an N–C long-bond and short-bond complex with a bond distance of 3.016 and 1.496 Å, and the corresponding binding energies (BE, without zero-point energy correction) are −3.0 and −12.0 kcal mol^–1^, characteristics of a van der Waals (vdW) complex and a covalent/dative bond (DB) for the former and latter. These two distinct minima are separated by a low threshold barrier (Δ*E*^‡^) of 3.5 kcal mol^–1^ from the vdW complex, with a corresponding reaction energy (Δ*E*_r_) of −9.1 kcal mol^–1^, in close agreement with the reported DFT and DLPNO–CCSD(T) computations,^23^ indicating the suitability of our selected level of theory. As mentioned above, inclusion of implicit solvent was shown to stabilize the C_18_–pip, and indeed, upon transitioning our computations to the solvent phase, the threshold barrier (2.2 kcal mol^–1^) is significantly lowered and the reaction energy (−17.7 kcal mol^–1^) becomes more exergonic, indicating the extra stability of the transition state and the DB complex exerted by the solvent as compared to the vdW adduct. Figure 1 depicts the N–C bond distances and the energetics of the double-well potential profile of C_18_–pip in the pip solvent.

Considering the low threshold barrier and high exergonicity of the above reaction, we embarked on investigating accurate QMT rates of this chemical transformation. Our computed tunneling rate constants reveal that the reaction is wholly dominated by ground-state QMT near absolute zero. In fact, at 4 K, the calculation shows that the classical over-the-barrier process is impossible, with a *k*_SC_ of ∼7 × 10^–110^ s^–1^. However, the assistance of QMT speeds up the reaction with a *k*_T_ of ∼9 × 10^–6^ s^–1^, which should lead to an experimentally observable rate. It can also be seen from Table 1 that as the temperature increases toward the 30 K sublimation temperature of the argon matrix, the tunneling rate increases rapidly, yielding a *k*_T_ of 0.02 s^–1^, corresponding to a half-life *t*_1/2_ (calculated as *t*_1/2_ = ln 2/*k*) of ∼38 s. This means that if we can observe the transformation of vdW → DB in an argon matrix doped with pip solvent, the vdW adduct will have a fleeting existence, and only the DB complex will be detected and isolated under matrix isolation spectroscopy. The transient behavior of the vdW complex can likewise be attributed to a similar case of quantum tunneling instability.^54^ Noteworthy, our gas-phase reaction rates also reveal that QMT completely drives this reaction at low temperatures (see the Supporting Information for the rates table) as well as that at the liquid N_2_ temperature (77 K) the vdW adduct (*t*_1/2_ = 0.6 s) will have a fleeting existence. However, its *k*_T_ rates are slower compared to those in solvent, as expected due to the higher barrier and low exergonicity of the reaction (*vide supra*).

**Table 1:** Semiclassical (*k*_SC_) and Tunneling-Corrected (*k*_T_) Rate Constants (in s^–1^) and Half-Lives (*t*_1/2_) (in s) for LB to SB Transformation in C_18_–Pip Adducts from 4 to 30 K

*T*	*k*_SC_	*k*_T_	*t*_1/2_
4	7 × 10^–110^	9 × 10^–6^	8 × 10^4^
6	10^–69^	9 × 10^–6^	8 × 10^4^
8	10^–49^	10^–5^	7 × 10^4^
10	10^–37^	2 × 10^–5^	5 × 10^4^
20	2 × 10^–13^	4 × 10^–4^	4 × 10^4^
30	2 × 10^–5^	0.02	38

**Figure 2:**
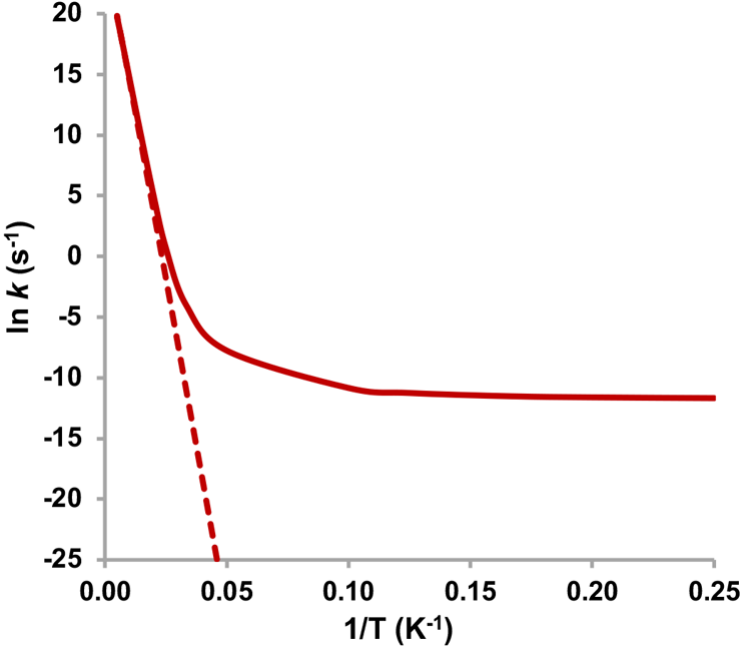
Arrhenius plot of the *k*_SC_ (dashed line) or *k*_T_ (solid line) against the inverse of temperature for a temperature range of 4–200 K.

The Arrhenius plot depicted in Figure 2 clearly shows a divergence of the tunneling-corrected *k*_T_ rates (curved line) from the semiclassical *k*_SC_ rate constants (straight line) and reaching a plateau as the temperature is lowered, further strengthening the case for QMT in the studied system.

Up to about 50 K, the role of QMT still dominates the overall reaction rate, but in this regime, excited vibrational levels of the molecules acquire significant population and QMT occurs from there by a thermally activated tunneling process.^55^ Note that as opposed to hydrogen-tunneling-based reactions where the vibrational energy levels are well-separated, the energy levels in the case of heavy-atom tunneling are compact and closely spaced, making the molecules easily accessible to higher vibrational energy levels even with a small amount of heat; therefore, we see a softer concave Arrhenius graph in the thermally activated tunneling regime.

We further analyzed the kinetic isotope effect (KIE), a standard probe for the “fingerprint” of QMT in a chemical reaction. Our computed tunneling-corrected KIEs of the atoms involved along the reaction axis, i.e., nitrogen and carbon of the N–C bond (KIE calculated as ^12^C/^13^C for carbon and ^14^N/^15^N for nitrogen) gives a maximum KIE of 1.41 and 2.99 at 4 K, respectively, indicating a clear case of heavy-atom QMT; however, among them the exceptionally higher KIE value of nitrogen (possibly a record for this atom) suggests that it is the “tunneling-determining atom”,^56^ i.e., the atom with the most dominant influence on the tunneling rates. As the reaction involves significant deformation of the C_18_ ring during the formation of the DB complex, we also computed the KIE on the ring carbons by substituting all of the 18 carbons by their heavier isotope in order to preserve symmetry during the reaction progress. This yields a surprisingly large KIE of 2.58 at 4 K, reflecting that all the carbons participate in the tunneling process. Furthermore, we also computed the KIE of hydrogen (H/D) attached to the nitrogen, which exhibits a large displacement during the reaction progress, and obtained a value of 2.21 at 4 K, giving an indication of hydrogen tunneling. However, because of its small atomic mass and the large mass disparity with its isotopologues (H/D), the KIE cannot be compared to the ratio of heavier atom counterparts, but it is clear that within the heavy atoms the nitrogen of the pip has a more decisive influence on the overall QMT rate.

We have also explored the double-well potentials of the complexes formed by analogues of C_18_, namely, C_14_ to C_22_ (see Table 2). All these systems considered here are closed-shell singlet ground states.^23,57,58^ The N–C bond lengths and its binding energy (BE) strength of the vdW form of these complexes are almost invariant except for C_14_–pip, which has the smallest BE. On the other hand, the bond distance of the DB complexes increases with increasing cyclo[*n*]carbon ring size, and their corresponding BEs decrease. The threshold barrier for the vdW → DB conversion is predicted to be lower with decreasing ring size and high exergonicity of the reaction showing Hammond’s postulate type correlation.^24^ This trend in the Δ*E*^‡^ and Δ*E*_r_ is due to the fact that in going from C_14_ to C_22_, what Hobza termed the “strain energy”, i.e., the energy required for ring deformation during complexation, increases, thereby explaining the higher Δ*E*^‡^ and destabilizing Δ*E*_r_.^23^ Notably, we have also studied smaller complexes formed by C_10_ and C_12_; however, it exhibits a barrierless flat potential yielding a single minimum-energy configuration with an N–C bond distance of 1.496 and 1.487 Å and BE of −39.4 and −37.5 kcal mol^–1^ for the former and latter, respectively.

**Table 2:** N–C Bond Distance for van der Waals (vdW), Dative Bond (DB), and Transition State (TS) Structures in Å, Their Respective Binding Energies (BE) along with Threshold Energies (from LB to TS, Δ*E*^‡^), Reaction Energies (Δ*E*_r_) in kcal mol^–1^, Transition State Imaginary Frequencies (υ) in cm^–1^, and Semiclassical (*k*_SC_) and Tunneling (*k*_T_) Rates in s^–1^ at 4 K for the Studied C_*n*_–Pip Complexes

system	vdW	DB	TS	BE_vdW_	BE_DB_	Δ*E*^‡^	Δ*E*_r_	υ	*k*_SC_	*k*_T_
C_14_–pip	2.914	1.489	2.536	–1.8	–29.0	0.1	–27.2	115*i*	6 × 10^5^	7 × 10^9^
C_16_–pip	2.934	1.501	2.380	–2.3	–23.5	1.2	–21.2	150*i*	4 × 10^–56^	100
C_18_–pip	3.016	1.496	2.307	–2.1	–19.7	2.2	–17.7	164*i*	7 × 10^–110^	9 × 10^–6^
C_20_–pip	2.989	1.504	2.241	–2.1	–17.0	2.8	–14.9	164*i*	2 × 10^–142^	2 × 10^–7^
C_22_–pip	3.016	1.505	2.207	–2.0	–14.6	3.5	–12.6	207*i*	5 × 10^–182^	9 × 10^–12^

Turning next to the QMT rates for the donor–acceptor complexes of these smaller analogues, our computations predict that the reactions are forbidden classically at 4 K except for C_14_, for which the reaction is almost barrierless, making the reaction feasible (see Table 2). However, the inclusion of tunneling correction in the overall rate constant accelerates the reaction by several orders of magnitude in all the studied cases. As shown in Table 2, the *k*_T_ is extremely fast for the complexes with smaller C_14_ and C_16_ ring sizes, corresponding to a short half-live of 0.1 ns and 0.01 s at 4 K for the former and latter, suggesting that the vdW long-bond adduct is fleetingly stable. For larger C_20_ and C_22_ systems, the QMT rate is lower than for their smaller analogues (Table 2) with extremely more prolonged *t*_1/2_ ranging from months to millennia at 4 K, indicating that only the vdW adduct will be isolable in a standard experimental setup. However, it should be mentioned that as the temperature is raised to 50 K, the role of thermally activated tunneling enhances the overall reaction rate in all the above systems, making the vdW complex’s existence fleeting (see the Supporting Information for the full rates table). For instance, the *k*_T_ for C_20_–pip and C_22_–pip results in a value of 0.7 and 5 × 10^–4^ s^–1^ at 50 K with *t*_1/2_ of 0.9 s and ∼20 min. The slow QMT rates for the larger systems can be attributed to the higher threshold barrier and less exergonicity of the reaction, which can produce a wider barrier width.

**Figure 3:**
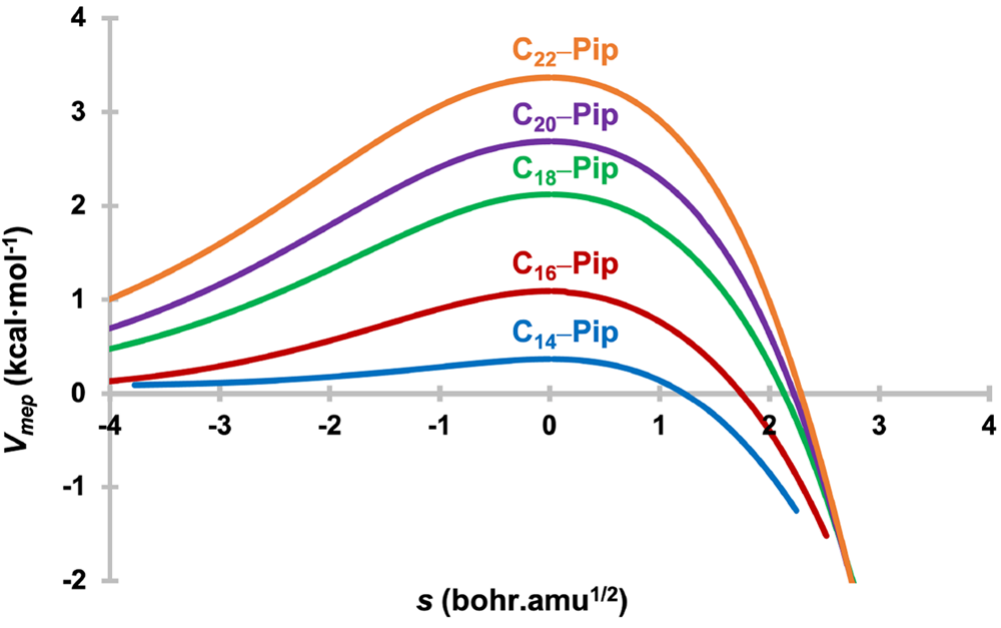
Minimum-energy-path potential (*V*_mep_) as a function of the reaction coordinate (*s*) in mass-scaled coordinates for the studied C_*n*_–pip complexes.

Because an accurate estimation of the barrier width (*w*) is difficult to determine—as real reactions do not exhibit a simple square or inverse parabolic PES, but instead run through a roughly Gaussian-shaped minimum-energy path (MEP)^25,59^—we therefore considered the potential energy profiles of the MEP (*V*_mep_) with respect to the mass-scaled reaction coordinate (*s*) (see Figure 3) and the changes of the ground state vibrational adiabatic potential energy curve [*V*_a_^G^(*s*)] along the reaction coordinate (*s*) (see Figure S1 in the Supporting Information) as a visual indicator of the influence of *w* on the tunneling rate differences in C_*n*_–pip systems. It can be seen clearly from Figure 3 that with the increase in the size of the cyclocarbon ring *w* gets wider along with higher Δ*E*^‡^, which nicely explains the reason for the decreasing QMT rates from C_14_–pip to C_22_–pip.

## Conclusion

In summary, our rate constant calculations including multidimensional tunneling correction reveal the overwhelming role of heavy atom QMT in the vdW → DB transformation of the C_18_–pip complex at cryogenic temperatures where the classical over-the-barrier process is virtually nonexistent. At the sublimation temperature of the argon matrix (30 K), the decay QMT rate is predicted to be extremely fast with a *t*_1/2_ of only 38 s, indicating the fleeting existence of vdW complex. The concave Arrhenius graph and the KIE analysis further sharpen the fingerprint of QMT in this reaction. Additionally, we explored the role of QMT in other C_*n*_–pip systems (*n* = 14, 16, 20, and 22) and predicted a rapid QMT rate for the smaller analogues of C_18_, with very to extremely short *t*_1/2_ ranging between seconds and nanoseconds at 4 K. The present study shows that QMT can play a determining role in the covalent/dative bond formation of C_18_–pip. Considering the recent work on the enhanced stability of C_60_ with pip, it remains to be tested if tunneling can act a key player in the covalent/dative bond functionalization of fullerenes.

## Supporting Information Available

Table of CVT and SCT rate constants as a function of temperature; polyrate input and output details; vibrational adiabatic potential energy profile; all relevant geometries in XMol/Molden.XYZ format; full references of Gaussian 16 and Polyrate17 (PDF)
