# Supplementary material for: Heavy-Atom Tunneling in the Covalent/Dative Bond Complexation of Cyclo[18]carbon–Piperidine
Source: J Phys Chem B. 2022 Feb 18;126(8):1799–804. doi: 10.1021/acs.jpcb.2c00218 (PMC8900127; doi:10.1021/acs.jpcb.2c00218)
Supplement: Supplementary file 1 — jp2c00218_si_001.pdf [file jp2c00218_si_001.pdf]

Heavy-Atom Tunneling in the Covalent/Dative Bond Complexation of Cyclo[18]carbon–piperidine

Ashim Nandi and Jan M. L. Martin**

Department of Molecular Chemistry and Materials Science, Weizmann Institute of Science, 7610001 Reḥovot, Israel. Email: ashim.nandi@weizmann.ac.il, gershom@weizmann.ac.il

Supporting Information

- Complete CVT and CVT + SCT rate constants from 4 K – 400 K for C_n–pip complexes.
- Ground state vibrational adiabatic potential energy curve [$V_a^G(s)$] profile
- Example of POLYRATE input file.
- XYZ coordinates for optimized structures.
- Full Gaussian 16 and POLYRATE references.

Complete set of POLYRATE. fu15 output files including CVT and SCT rate constants in s^{-1} at different temperatures for C_n–pip complexes.

C_{18}–pip			C_{14}–pip			C_{16}–pip		
T(K)	CVT	CVT/SCT	T(K)	CVT	CVT/SCT	T(K)	CVT	CVT/SCT
4	7.24E-110	8.51E-06	4	5.50E+05	6.54E+09	4	4.25E-56	1.26E+02
5	8.74E-86	9.08E-06	5	7.41E+06	8.71E+09	5	8.94E-43	1.24E+02
6	1.00E-69	9.93E-06	6	4.33E+07	1.11E+10	6	6.87E-34	1.24E+02
8	1.20E-49	1.30E-05	8	4.17E+08	1.70E+10	8	8.80E-23	1.29E+02
10	1.34E-37	1.96E-05	10	1.70E+09	2.41E+10	10	4.04E-16	1.42E+02
20	1.66E-13	4.27E-04	20	3.38E+10	7.64E+10	20	8.04E-03	6.62E+02
30	1.73E-05	1.82E-02	30	9.74E+10	1.39E+11	30	2.06E+02	1.08E+04
40	1.71E-01	2.04E+00	40	1.63E+11	1.91E+11	40	3.16E+04	2.04E+05
50	4.15E+01	1.47E+02	50	2.18E+11	2.28E+11	50	6.34E+05	1.85E+06
75	5.94E+04	9.24E+04	75	3.01E+11	2.75E+11	75	3.23E+07	4.88E+07
77.36	9.21E+04	1.39E+05	77.36	3.06E+11	2.76E+11	77.36	4.09E+07	5.98E+07
100	2.15E+06	2.61E+06	100	3.31E+11	2.72E+11	100	2.21E+08	2.60E+08
125	1.80E+07	1.93E+07	125	3.33E+11	2.55E+11	125	6.77E+08	6.97E+08
150	7.31E+07	7.22E+07	150	3.22E+11	2.31E+11	150	1.40E+09	1.32E+09
175	1.96E+08	1.84E+08	175	3.06E+11	2.09E+11	175	2.30E+09	2.02E+09
194.7	3.54E+08	3.18E+08	194.7	2.92E+11	1.93E+11	194.7	3.07E+09	2.55E+09
200	4.07E+08	3.62E+08	200	2.88E+11	1.88E+11	200	3.28E+09	2.69E+09
225	7.09E+08	6.04E+08	225	2.70E+11	1.71E+11	225	4.27E+09	3.35E+09
250	1.08E+09	7.49E+08	250	2.53E+11	1.55E+11	250	5.21E+09	3.92E+09
273.15	1.47E+09	1.05E+09	273.15	2.38E+11	1.43E+11	273.15	6.02E+09	4.43E+09
275	1.51E+09	1.07E+09	275	2.37E+11	1.42E+11	275	6.08E+09	4.47E+09
298.15	1.94E+09	1.41E+09	298.15	2.24E+11	1.33E+11	298.15	6.82E+09	4.94E+09
300	1.98E+09	1.44E+09	300	2.23E+11	1.33E+11	300	6.87E+09	4.98E+09
325	2.49E+09	1.85E+09	325	2.10E+11	1.22E+11	325	7.58E+09	5.30E+09
350	3.04E+09	2.29E+09	350	1.98E+11	1.13E+11	350	8.18E+09	5.48E+09
373.15	3.54E+09	2.58E+09	373.15	1.88E+11	1.06E+11	373.15	8.64E+09	5.48E+09
375	3.58E+09	2.60E+09	375	1.88E+11	1.05E+11	375	8.67E+09	5.48E+09
400	4.12E+09	2.89E+09	400	1.78E+11	9.81E+10	400	9.07E+09	5.66E+09

C_{20}–pip

T(K)	CVT	CVT/SCT
4	2.06E-142	1.51E-07
5	8.34E-112	1.55E-07
6	2.16E-91	1.60E-07
8	7.23E-66	1.77E-07
10	1.51E-50	2.05E-07
20	6.66E-20	1.09E-06
30	1.06E-09	2.65E-05
40	1.28E-04	3.66E-03
50	1.40E-01	6.95E-01
75	1.47E+03	2.55E+03
77.36	2.57E+03	4.28E+03
100	1.42E+05	1.82E+05
125	2.15E+06	2.37E+06
150	1.29E+07	1.28E+07
175	4.53E+07	4.23E+07
194.7	9.64E+07	8.64E+07
200	1.15E+08	1.02E+08
225	2.35E+08	2.00E+08
250	4.12E+08	3.41E+08
273.15	6.28E+08	5.08E+08
275	6.48E+08	5.23E+08
298.15	9.15E+08	7.20E+08
300	9.39E+08	7.37E+08
325	1.28E+09	9.74E+08
350	1.65E+09	1.23E+09
373.15	2.03E+09	1.48E+09
375	2.06E+09	1.50E+09
400	2.49E+09	1.77E+09

C_{22}–pip

T(K)	CVT	CVT/SCT
4	4.63E-182	9.36E-12
5	1.57E-143	9.71E-12
6	7.77E-118	1.02E-11
8	1.03E-85	1.16E-11
10	1.95E-66	1.40E-11
20	6.86E-28	1.43E-10
30	4.64E-15	6.70E-09
40	1.15E-08	1.27E-06
50	7.68E-05	5.90E-04
75	8.96E+00	1.75E+01
77.36	1.82E+01	3.37E+01
100	2.91E+03	3.95E+03
125	9.05E+04	1.04E+05
150	8.78E+05	9.15E+05
175	4.39E+06	4.28E+06
194.7	1.15E+07	1.08E+07
200	1.45E+07	1.34E+07
225	3.63E+07	3.24E+07
250	7.52E+07	6.52E+07
273.15	1.30E+08	1.11E+08
275	1.36E+08	1.16E+08
298.15	2.13E+08	1.78E+08
300	2.20E+08	1.83E+08
325	3.31E+08	2.68E+08
350	4.66E+08	3.69E+08
373.15	6.13E+08	4.76E+08
375	6.26E+08	4.85E+08
400	8.05E+08	5.91E+08

C_{18}–pip (gas-phase)

T(K)	CVT	CVT/SCT
4	4.63E-200	2.41E-12
5	5.88E-158	2.88E-12
6	6.87E-130	3.27E-12
8	8.18E-95	3.85E-12
10	8.87E-74	4.26E-12
20	9.03E-32	6.07E-12
30	8.01E-18	1.81E-11
40	6.98E-11	3.83E-09
50	9.69E-07	5.58E-06
75	2.93E-01	5.33E-01
77.36	6.28E-01	1.09E+00
100	1.50E+02	2.00E+02
125	6.11E+03	7.02E+03
150	7.06E+04	7.43E+04
175	3.98E+05	3.93E+05
194.7	1.13E+06	1.07E+06
200	1.44E+06	1.35E+06
225	3.85E+06	3.48E+06
250	8.38E+06	7.33E+06
273.15	1.51E+07	1.28E+07
275	1.57E+07	1.33E+07
298.15	2.54E+07	2.09E+07
300	2.63E+07	2.16E+07
325	4.05E+07	3.24E+07
350	5.82E+07	4.53E+07
373.15	7.76E+07	5.90E+07
375	7.93E+07	6.01E+07
400	1.03E+08	7.65E+07

C_{18}–pip (^{13}C on the C-N bond)

T(K)	CVT	CVT/SCT	KIE(SCT)
4	3.16E-110	6.04E-06	1.41
5	4.50E-86	6.40E-06	1.42
6	5.76E-70	6.94E-06	1.43
8	7.91E-50	8.91E-06	1.46
10	9.60E-38	1.32E-05	1.48
20	1.41E-13	2.96E-04	1.44
30	1.55E-05	1.37E-02	1.33
40	1.57E-01	1.72E+00	1.19
50	3.89E+01	1.30E+02	1.13
75	5.68E+04	8.54E+04	1.08
77.36	8.82E+04	1.28E+05	1.09
100	2.08E+06	2.46E+06	1.06
125	1.76E+07	1.84E+07	1.05
150	7.15E+07	6.93E+07	1.04
175	1.92E+08	1.78E+08	1.03
194.7	3.48E+08	3.08E+08	1.03
200	3.99E+08	3.51E+08	1.03
225	6.98E+08	5.87E+08	1.03
250	1.06E+09	7.36E+08	1.02
273.15	1.45E+09	1.03E+09	1.02
275	1.48E+09	1.06E+09	1.01
298.15	1.92E+09	1.39E+09	1.01
300	1.95E+09	1.42E+09	1.01
325	2.46E+09	1.83E+09	1.01
350	3.00E+09	2.27E+09	1.01
373.15	3.51E+09	2.54E+09	1.02
375	3.55E+09	2.56E+09	1.02
400	4.09E+09	2.85E+09	1.01

C_{18}–pip (^{13}C on all ring carbons)

T(K)	CVT	CVT/SCT	KIE (SCT)
4	1.05E-110	3.30E-06	2.58
5	1.86E-86	3.51E-06	2.59
6	2.76E-70	3.83E-06	2.59
8	4.54E-50	5.02E-06	2.59
10	6.15E-38	7.83E-06	2.50
20	1.12E-13	2.10E-04	2.03
30	1.33E-05	1.13E-02	1.61
40	1.40E-01	1.52E+00	1.34
50	3.52E+01	1.19E+02	1.24
75	5.29E+04	8.15E+04	1.13
77.36	8.23E+04	1.23E+05	1.13
100	1.96E+06	2.37E+06	1.10
125	1.67E+07	1.78E+07	1.08
150	6.84E+07	6.77E+07	1.07
175	1.85E+08	1.73E+08	1.06
194.7	3.35E+08	3.01E+08	1.06
200	3.85E+08	3.42E+08	1.06
225	6.73E+08	5.70E+08	1.06
250	1.04E+09	7.09E+08	1.06
273.15	1.41E+09	9.92E+08	1.06
275	1.44E+09	1.02E+09	1.05
298.15	1.86E+09	1.34E+09	1.05
300	1.90E+09	1.37E+09	1.05
325	2.39E+09	1.76E+09	1.05
350	2.92E+09	2.19E+09	1.05
373.15	3.41E+09	2.47E+09	1.04
375	3.45E+09	2.50E+09	1.04
400	3.97E+09	2.78E+09	1.04

C_{18}–pip (^{15}N)

T(K)	CVT	CVT/SCT	KIE (SCT)
4	1.15E-109	2.85E-06	2.99
5	1.26E-85	3.13E-06	2.90
6	1.36E-69	3.58E-06	2.77
8	1.51E-49	5.50E-06	2.36
10	1.61E-37	1.02E-05	1.92
20	1.82E-13	3.59E-04	1.19
30	1.83E-05	1.67E-02	1.09
40	1.78E-01	2.01E+00	1.01
50	4.28E+01	1.48E+02	0.99
75	6.04E+04	9.39E+04	0.98
77.36	9.35E+04	1.41E+05	0.99
100	2.17E+06	2.64E+06	0.99
125	1.81E+07	1.94E+07	0.99
150	7.33E+07	7.25E+07	1.00
175	1.96E+08	1.84E+08	1.00
194.7	3.54E+08	3.18E+08	1.00
200	4.06E+08	3.61E+08	1.00
225	7.07E+08	6.02E+08	1.00
250	1.07E+09	7.50E+08	1.00
273.15	1.46E+09	1.05E+09	1.00
275	1.49E+09	1.07E+09	1.00
298.15	1.93E+09	1.41E+09	1.00
300	1.96E+09	1.44E+09	1.00
325	2.47E+09	1.85E+09	1.00
350	3.02E+09	2.20E+09	1.04
373.15	3.51E+09	2.55E+09	1.01
375	3.55E+09	2.57E+09	1.01
400	4.08E+09	2.85E+09	1.01

C_{18}–pip (D on N-H)

T(K)	CVT	CVT/SCT	KIE(SCT)
4	3.01E-109	3.85E-06	2.21
5	2.73E-85	4.20E-06	2.16
6	2.59E-69	4.76E-06	2.09
8	2.44E-49	7.19E-06	1.81
10	2.36E-37	1.33E-05	1.47
20	2.20E-13	4.38E-04	0.97
30	2.09E-05	1.87E-02	0.97
40	1.97E-01	2.19E+00	0.93
50	4.64E+01	1.60E+02	0.92
75	6.39E+04	1.00E+05	0.92
77.36	9.89E+04	1.50E+05	0.93
100	2.27E+06	2.77E+06	0.94
125	1.88E+07	2.02E+07	0.96
150	7.54E+07	7.47E+07	0.97
175	2.00E+08	1.86E+08	0.99
194.7	3.60E+08	3.25E+08	0.98
200	4.12E+08	3.68E+08	0.98
225	7.15E+08	6.10E+08	0.99
250	1.09E+09	7.52E+08	1.00
273.15	1.47E+09	1.05E+09	1.00
275	1.51E+09	1.07E+09	1.00
298.15	1.94E+09	1.41E+09	1.00
300	1.97E+09	1.44E+09	1.00
325	2.48E+09	1.84E+09	1.01
350	3.01E+09	2.18E+09	1.05
373.15	3.50E+09	2.50E+09	1.03
375	3.54E+09	2.53E+09	1.03
400	4.06E+09	2.85E+09	1.01

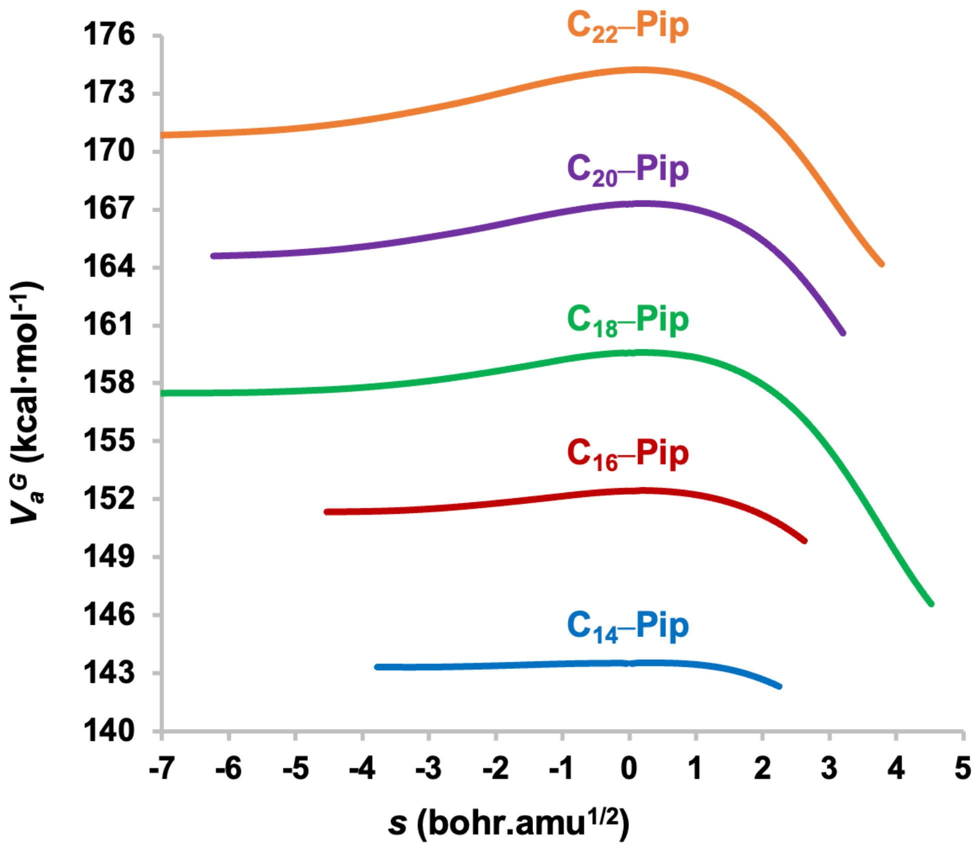

Fig S1. Profile of the ground state vibrational adiabatic potential energy curve [V_a^G(s), in kcal mol^{-1}] along the reaction coordinate (*s*) in mass-scaled coordinates (Bohr-amu$^{1/2}$) for the studied C_n–pip complexes. The profile for V_a^G(s) is obtained by correcting the V_{mep} curve by including zero point vibrational energy (ZPE) as $V_a^G(s) = V_{mep}(s) + ZPVE(s)$.

Example of POLYRATE input file.

```
*GENERAL

TITLE
  QMT in c18_pip
END

# DL ISPE

ATOMS
1 C
2 C
3 C
4 C
5 C
6 C
7 C
8 C
9 C
10 C
11 C
12 C
13 C
14 C
15 C
16 C
17 C
18 C
19 C
20 C
21 C
22 C
23 C
24 H
25 H
26 H
27 H
28 H
29 H
30 H
31 H
32 H
33 H
34 N
35 H
END

NOSUPERMOL

*SECOND

HESSCAL hhook

FPRINT

*OPTIMIZATION

PRINT

OPTMIN ohook
OPTTS ohook

*REACT1
INITGEO hooks
GEOM
1
2
3
4
5
6
7
8
9
10
11
12
13
14
15
16
17
18
19
20
21
22
23
24
25
26
27
28
29
30
31
32
33
34
35
END
SPECIES nonlinrp

*PROD1
INITGEO hooks
GEOM
1
2
3
4
5
6
7
8
9
10
11
12
13
14
15
16
17
18
19
20
21
22
23
24
25
26
27
28
29
30
31
32
33
34
35
END
SPECIES nonlinrp

*START
INITGEO hooks
GEOM
1
2
3
4
5
6
7
8
9
10
11
12
13
14
15
16
17
18
19
20
21
22
23
24
25
26
27
28
29
30
31
32
33
34
35
END
SPECIES nonlints
PROJECT

*PATH
#SYMMETRY
INTMU 3
SSTEP 0.002
RPM pagem
SRANGE
  SLP 3.8
  SLM -2.4
END
PRPATH
  coord 1 2
  xmol
  freq 99
END

*TUNNEL
ZCT
SCT
QRST
  harmonic
  mode 99
  states all
END

*RATE
FORWARDK
SIGMAF 1
TST
CVT
PRDELG
PRPART rtp

TEMP
2
3
4
5
6
8
10
20
30
40
50
75
77.355
100
125
150
175
194.7
200
225
250
273.15
275
298.15
300
325
350
373.15
375
400
END

ANALYSIS
2
3
4
5
6
8
10
20
30
40
50
75
77.355
100
125
150
175
194.7
200
225
250
273.15
275
298.15
300
325
350
373.15
375
400
END

EACT
2. 3.
3. 4
4. 6.
6. 10.
10. 20.
20. 50.
50. 100.
200. 225.
300. 325.
END

GTLOG
```

XYZ coordinates for optimized structures

C_{10}

C	0.000000	2.072116	-0.000000
C	1.217959	1.676377	0.000000
C	1.970700	0.640319	0.000000
C	1.970700	-0.640319	0.000000
C	-1.217959	1.676377	-0.000000
C	-1.970700	0.640319	-0.000000
C	-1.970700	-0.640319	-0.000000
C	-1.217959	-1.676377	-0.000000
C	-0.000000	-2.072116	-0.000000
C	1.217959	-1.676377	0.000000

C_{12}

C	-2.569970	-0.076960	0.000000
C	-2.070534	1.195274	0.000000
C	-1.351469	2.186995	0.000000
C	0.000003	2.390758	0.000000
C	1.218408	2.264074	-0.000000
C	-2.070534	-1.195501	0.000000
C	-1.218408	-2.264074	0.000000
C	-0.000003	-2.390758	0.000000
C	1.351469	-2.186995	0.000000
C	2.070534	-1.195274	0.000000
C	2.569970	0.076960	0.000000
C	2.070534	1.195501	0.000000

C_{14}

C	0.618426	2.812870	0.000000
C	-0.618426	2.812870	-0.000000
C	-1.813608	2.237300	0.000000
C	-2.584772	1.270291	0.000000
C	-2.879958	-0.023002	0.000000
C	1.813608	2.237300	-0.000000
C	2.584772	1.270291	0.000000
C	2.879958	-0.023002	0.000000
C	2.604732	-1.228843	0.000000
C	1.777641	-2.265983	-0.000000
C	0.663276	-2.802633	0.000000
C	-0.663276	-2.802633	0.000000
C	-1.777641	-2.265983	0.000000
C	-2.604732	-1.228843	-0.000000

C_{16}

C	0.607042	3.249707	0.000000
C	-0.607042	3.249707	-0.000000
C	-1.868647	2.727134	0.000000
C	-2.727134	1.868647	-0.000000
C	-3.249707	0.607042	0.000000
C	-3.249707	-0.607042	-0.000000
C	1.868647	2.727134	-0.000000
C	2.727134	1.868647	0.000000
C	3.249707	0.607042	-0.000000
C	3.249707	-0.607042	0.000000
C	2.727134	-1.868647	-0.000000
C	1.868647	-2.727134	0.000000
C	0.607042	-3.249707	-0.000000
C	-0.607042	-3.249707	0.000000
C	-1.868647	-2.727134	-0.000000
C	-2.727134	-1.868647	0.000000

C_{18}

C	0.611551	3.646377	0.000000
C	-0.611551	3.646377	-0.000000
C	-1.875371	3.186384	0.000000
C	-2.812321	2.400189	0.000000
C	-3.484786	1.235447	0.000000
C	-3.697175	0.030926	-0.000000
C	-3.463631	-1.293570	0.000000
C	1.875371	3.186384	-0.000000
C	2.812321	2.400189	0.000000
C	3.484786	1.235447	0.000000
C	3.697175	0.030926	0.000000
C	3.463631	-1.293570	-0.000000
C	2.852079	-2.352807	0.000000
C	1.821804	-3.217311	0.000000
C	0.672464	-3.635636	0.000000
C	-0.672464	-3.635636	-0.000000
C	-1.821804	-3.217311	0.000000
C	-2.852079	-2.352807	0.000000

C_{20}

C	0.607653	4.068712	0.000000
C	-0.607653	4.068712	-0.000000
C	-1.899927	3.648827	-0.000000
C	-2.883130	2.934488	-0.000000
C	-3.681800	1.835213	-0.000000
C	-4.057350	0.679389	-0.000000
C	4.057350	-0.679389	-0.000000
C	3.681800	-1.835213	-0.000000
C	2.883130	-2.934488	-0.000000
C	1.899927	-3.648827	-0.000000
C	0.607653	-4.068712	-0.000000
C	-0.607653	-4.068712	0.000000
C	-1.899927	-3.648827	-0.000000
C	4.057350	0.679389	-0.000000
C	3.681800	1.835213	-0.000000
C	-2.883130	-2.934488	-0.000000
C	1.899927	3.648827	-0.000000
C	-4.057350	-0.679389	0.000000
C	-3.681800	-1.835213	-0.000000
C	2.883130	2.934488	-0.000000

C_{22}

C	0.609553	4.471249	0.000000
C	−0.609553	4.471249	−0.000000
C	−1.904551	4.091003	0.000000
C	−2.930128	3.431905	0.000000
C	−3.813974	2.411893	0.000000
C	−4.320409	1.302955	−0.000000
C	4.512486	−0.032976	−0.000000
C	4.338990	−1.239674	0.000000
C	3.778317	−2.467376	0.000000
C	2.979972	−3.388715	0.000000
C	1.844558	−4.118401	−0.000000
C	0.674835	−4.461863	0.000000
C	−0.674835	−4.461863	0.000000
C	−4.512486	−0.032976	0.000000
C	4.320409	1.302955	0.000000
C	3.813974	2.411893	0.000000
C	−1.844558	−4.118401	0.000000
C	−2.979972	−3.388715	−0.000000
C	1.904551	4.091003	−0.000000
C	−4.338990	−1.239674	0.000000
C	−3.778317	−2.467376	0.000000
C	2.930128	3.431905	0.000000

C_{14}–pip

vdW

C	−0.400260	−0.013028	−0.813230
C	−0.210286	1.206554	−0.707506
C	0.610956	2.217398	−0.458544
C	1.649743	2.827230	−0.172092
C	2.937773	2.812561	0.144744
C	4.060279	2.358861	0.401736
C	−0.082577	−1.301205	−0.792763
C	0.735843	−2.214061	−0.631872
C	1.902643	−2.790325	−0.369099
C	3.101129	−2.725429	−0.070706
C	4.261910	−2.162697	0.242210
C	4.946335	−1.154841	0.457058
C	5.228970	0.135386	0.587001
C	4.870881	1.319323	0.553139
C	−3.756048	0.877952	−0.794207
C	−5.118747	1.022073	−0.117246
C	−5.093617	0.387704	1.271746
C	−4.610052	−1.058841	1.189159
C	−3.263598	−1.126147	0.471013
H	−5.395489	2.077042	−0.056719
H	−3.013580	1.461650	−0.237030
H	−3.780682	1.277182	−1.809516
H	−4.409959	0.956195	1.911757
H	−6.079987	0.438314	1.736220
H	−4.524355	−1.496967	2.186073
H	−5.340185	−1.656984	0.633731
H	−2.509476	−0.606091	1.073885
H	−2.928074	−2.159108	0.360867
H	−5.875451	0.523325	−0.732077
N	−3.273007	−0.498067	−0.847821
H	−3.859370	−1.038887	−1.475688

TS

C	−0.518196	0.014869	−0.551682
C	−0.281850	1.238169	−0.458839
C	0.631313	2.168252	−0.269864
C	1.687868	2.796595	−0.068715
C	2.989250	2.723945	0.140812
C	4.151238	2.316508	0.314871
C	−0.158770	−1.263373	−0.547336
C	0.698440	−2.150272	−0.443741
C	1.862212	−2.763850	−0.278026
C	3.082726	−2.699830	−0.074795
C	4.293914	−2.209037	0.145583
C	4.986222	−1.191670	0.302104
C	5.337246	0.080087	0.414248
C	4.935539	1.255280	0.397735
C	−3.521824	1.089381	−0.458981
C	−5.019438	1.142356	−0.160070
C	−5.366815	0.193852	0.986052
C	−4.861474	−1.217022	0.688922
C	−3.368944	−1.186960	0.366352
H	−5.313481	2.166017	0.080985
H	−2.965037	1.481450	0.400417
H	−3.266505	1.714966	−1.316238
H	−4.894196	0.555925	1.905446
H	−6.443488	0.185988	1.163488
H	−5.045911	−1.881769	1.535442
H	−5.403038	−1.626791	−0.170000
H	−2.811516	−0.867497	1.254618
H	−3.001937	−2.178616	0.095534
H	−5.572497	0.848580	−1.058166
N	−3.034766	−0.259616	−0.709244
H	−3.390983	−0.604642	−1.594597

DB

C	−1.151326	−0.347576	0.018208
C	−0.589784	0.903344	0.002401
C	0.365126	1.697225	−0.008045
C	1.473978	2.412006	−0.017269
C	2.722304	2.489861	−0.021050
C	4.032222	2.374481	−0.022898
C	−0.395553	−1.476926	0.022921
C	0.720773	−2.048362	0.022863
C	1.928227	−2.544658	0.022929
C	3.179130	−2.363230	0.017546
C	4.434183	−2.023735	0.011940
C	5.061263	−0.905408	0.001132
C	5.529684	0.294129	−0.009321
C	4.848868	1.405382	−0.017025
C	−3.242925	0.055725	−1.248854
C	−4.747128	−0.142486	−1.247942
C	−5.390580	0.511533	−0.030003
C	−4.750916	−0.017136	1.249268
C	−3.246948	0.182112	1.234473
H	−5.138641	0.278017	−2.174165
H	−2.969884	1.110668	−1.289781
H	−2.750059	−0.467893	−2.065568
H	−5.256127	1.595414	−0.084016
H	−6.462563	0.318256	−0.022025
H	−5.145513	0.493960	2.127340
H	−4.977290	−1.080381	1.371416
H	−2.976501	1.236398	1.168086
H	−2.755259	−0.253119	2.102116
H	−4.973480	−1.212684	−1.263238
N	−2.635487	−0.469100	0.024051
H	−2.829865	−1.472621	0.073831

C_{16}–pip

vdW

C	−1.107163	−2.997790	−0.429341
C	−2.274677	−3.264314	−0.228563
C	−3.596416	−3.021409	0.014665
C	−4.603019	−2.372097	0.213550
C	−5.367128	−1.252992	0.383705
C	−5.616473	−0.067327	0.464170
C	0.008248	−2.230103	−0.607605
C	0.659842	−1.209416	−0.697063
C	0.907959	0.133067	−0.704603
C	0.664685	1.320446	−0.622650
C	−0.120083	2.424228	−0.447536
C	−1.115030	3.091190	−0.247187
C	−2.438608	3.321729	−0.002136
C	−3.610283	3.075675	0.200853
C	−4.716437	2.293892	0.375102
C	−5.373792	1.276609	0.461688
C	4.301090	0.654543	−0.947135
C	5.686651	0.857540	−0.335209
C	5.656008	0.558698	1.162201
C	5.083720	−0.834429	1.416032
C	3.718300	−0.977020	0.745088
H	6.654282	0.649651	1.593939
H	6.395753	0.183230	−0.827116
H	6.029710	1.877991	−0.520097
H	3.610475	1.396309	−0.529165
H	4.324852	0.810746	−2.026777
H	5.762942	−1.588725	1.004809
H	4.995115	−1.028680	2.487267
H	3.321215	−1.985507	0.874002
H	3.010691	−0.288499	1.222666
H	5.023430	1.300676	1.661739
N	3.732048	−0.663028	−0.681387
H	4.262833	−1.370313	−1.179715

TS

C	-1.210060	-2.956968	-0.324890
C	-2.394751	-3.192528	-0.203496
C	-3.729917	-2.952527	-0.043957
C	-4.732015	-2.281260	0.096900
C	-5.497714	-1.158138	0.231343
C	-5.683647	0.040129	0.303850
C	-0.056579	-2.231184	-0.421798
C	0.682054	-1.269175	-0.462571
C	1.118774	0.031641	-0.453102
C	0.879426	1.232212	-0.371911
C	0.004316	2.261254	-0.232090
C	-0.966263	2.985972	-0.092121
C	-2.301440	3.207307	0.064597
C	-3.503376	3.069472	0.192567
C	-4.645256	2.328766	0.286812
C	-5.397365	1.374996	0.329991
C	4.032884	0.875304	-0.756647
C	5.542764	0.908573	-0.525094
C	5.886415	0.346265	0.852483
C	5.285353	-1.046824	1.026662
C	3.782835	-1.010361	0.758539
H	6.967844	0.317587	0.994368
H	6.035568	0.309751	-1.297575
H	5.906351	1.932796	-0.628391
H	3.538616	1.562335	-0.058798
H	3.778615	1.206308	-1.764644
H	5.757274	-1.740792	0.323725
H	5.470647	-1.428199	2.032751
H	3.343343	-2.007105	0.820643
H	3.292619	-0.388471	1.516187
H	5.480013	1.010173	1.622851
N	3.450013	-0.439495	-0.543449
H	3.700388	-1.075472	-1.293383

DB

C	-1.206024	-2.434068	-0.252562
C	-2.368701	-2.780777	-0.226425
C	-3.732958	-2.796246	-0.168767
C	-4.810458	-2.237994	-0.084638
C	-5.755232	-1.258150	0.023211
C	-5.866910	-0.044304	0.111973
C	-0.052550	-1.700043	-0.254581
C	0.835950	-0.876180	-0.241575
C	1.723395	0.237259	-0.220337
C	1.378977	1.513900	-0.108498
C	0.149530	2.039406	-0.011932
C	-0.945660	2.620267	0.078795
C	-2.274701	2.732036	0.147391
C	-3.507531	2.758068	0.203368
C	-4.670283	2.078318	0.207533
C	-5.609830	1.286760	0.192786
C	4.068852	0.922808	-0.790705
C	5.493116	0.410162	-0.907322
C	6.003271	-0.119914	0.428806
C	5.059692	-1.192047	0.962458
C	3.636994	-0.673339	1.053550
H	7.008193	-0.525031	0.315327
H	5.539921	-0.377025	-1.666120
H	6.113304	1.231778	-1.265495
H	3.983592	1.742697	-0.080003
H	3.657719	1.252389	-1.741958
H	5.083190	-2.071286	0.311870
H	5.359834	-1.524333	1.956263
H	2.931878	-1.444156	1.359705
H	3.549885	0.176081	1.731806
H	6.063406	0.702280	1.147241
N	3.169110	-0.162195	-0.283123
H	3.231663	-0.943563	-0.941942

C_{18}–pip

vdW

C	−0.401724	−3.141656	−0.788568
C	−1.502028	−3.583448	−0.489370
C	−2.798689	−3.604298	−0.129966
C	−3.912886	−3.211903	0.186351
C	−4.917931	−2.366560	0.479683
C	−5.523064	−1.320350	0.666058
C	−5.765368	0.000224	0.756152
C	0.589200	−2.268930	−1.048659
C	1.168730	−1.202072	−1.193385
C	1.382298	0.125838	−1.229604
C	1.161448	1.325868	−1.145611
C	0.482906	2.469561	−0.939213
C	−0.425203	3.243762	−0.671567
C	−1.651248	3.676110	−0.324081
C	−2.829251	3.663034	0.004091
C	−4.042043	3.181053	0.332214
C	−4.938852	2.383740	0.567946
C	−5.573774	1.207598	0.723637
C	4.796754	0.697996	−0.681212
C	5.983002	0.906420	0.259689
C	5.602241	0.526037	1.688942
C	5.039642	−0.893396	1.730721
C	3.885224	−1.035704	0.740191
H	6.461815	0.619848	2.354975
H	6.816575	0.279649	−0.074712
H	6.319636	1.944492	0.211105
H	3.996056	1.396318	−0.410758
H	5.075929	0.914308	−1.713652
H	5.827133	−1.606345	1.464125
H	4.701188	−1.147259	2.737813
H	3.506280	−2.059189	0.723860
H	3.057092	−0.391673	1.059940
H	4.839183	1.222976	2.052661
N	4.234008	−0.648032	−0.624394
H	4.904754	−1.310439	−1.001237

TS

C	−0.599568	−3.094792	−0.325685
C	−1.752010	−3.491049	−0.231869
C	−3.092641	−3.501698	−0.107908
C	−4.239702	−3.095287	0.011078
C	−5.280350	−2.250115	0.134415
C	−5.871237	−1.182824	0.222567
C	−6.089124	0.143979	0.284627
C	0.480376	−2.292849	−0.400714
C	1.198026	−1.305011	−0.437107
C	1.653489	−0.028090	−0.439801
C	1.390183	1.180580	−0.377862
C	0.587373	2.243926	−0.272295
C	−0.322437	3.062743	−0.161501
C	−1.589613	3.484013	−0.031119
C	−2.805476	3.585867	0.086041
C	−4.077794	3.165780	0.191269
C	−5.083429	2.471241	0.263880
C	−5.799539	1.332755	0.294745
C	4.497860	0.904904	−0.696764
C	6.010711	0.937602	−0.487218
C	6.378067	0.338706	0.868635
C	5.784794	−1.061053	1.014454
C	4.278126	−1.022609	0.772692
H	7.461724	0.309784	0.991414
H	6.493564	0.362959	−1.283776
H	6.368014	1.965928	−0.567574
H	4.010487	1.569641	0.026929
H	4.225847	1.260455	−1.691644
H	6.247160	−1.734187	0.285514
H	5.987076	−1.468824	2.006684
H	3.841178	−2.021527	0.813912
H	3.797669	−0.421406	1.552617
H	5.982730	0.979653	1.663696
N	3.926267	−0.418418	−0.509262
H	4.164179	−1.033877	−1.280304

DB

C	−0.605964	−2.740877	−0.214349
C	−1.764964	−3.132766	−0.185716
C	−3.100239	−3.273046	−0.140622
C	−4.274299	−2.922647	−0.082248
C	−5.424217	−2.235186	−0.010521
C	−6.001268	−1.149024	0.056689
C	−6.344547	0.141320	0.123315
C	0.518092	−2.000083	−0.229248
C	1.424670	−1.182276	−0.234506
C	2.219528	−0.031327	−0.231156
C	1.712485	1.197327	−0.148102
C	0.680122	1.955894	−0.070806
C	−0.323005	2.736514	0.003223
C	−1.577401	3.060363	0.067186
C	−2.807894	3.328527	0.124922
C	−4.056051	2.923408	0.155659
C	−5.204915	2.430913	0.177049
C	−5.888809	1.294245	0.155202
C	4.460694	0.912123	−0.820716
C	5.938164	0.577691	−0.913517
C	6.498790	0.161154	0.442124
C	5.693480	−1.004031	1.006442
C	4.217529	−0.662849	1.082131
H	7.547558	−0.117273	0.346514
H	6.086415	−0.223416	−1.643595
H	6.453897	1.457057	−1.298786
H	4.272556	1.740972	−0.139552
H	4.020522	1.150901	−1.786201
H	5.827992	−1.891457	0.380827
H	6.028240	−1.267883	2.009549
H	3.609711	−1.503907	1.410432
H	4.023858	0.190443	1.732929
H	6.448352	1.007566	1.132620
N	3.698020	−0.257915	−0.273662
H	3.854886	−1.049801	−0.903730

C_{20}–pip

vdW

C	1.003535	2.530593	−0.906556
C	0.152948	3.374703	−0.703742
C	−1.042872	3.959386	−0.429447
C	−2.216374	4.131244	−0.164233
C	−3.522412	3.890347	0.124185
C	−4.572009	3.321540	0.351581
C	0.216670	−3.259070	−0.802667
C	−0.827885	−3.837307	−0.575729
C	−2.134775	−4.073707	−0.287164
C	−3.310303	−3.916000	−0.022290
C	−4.503660	−3.326152	0.251520
C	−5.361759	−2.489858	0.454298
C	−5.987679	−1.293601	0.609421
C	1.148785	−2.289419	−0.998371
C	1.672142	−1.197787	−1.103287
C	−6.197196	−0.098130	0.671375
C	1.645986	1.343352	−1.064009
C	−5.493414	2.341443	0.544834
C	−6.014307	1.248281	0.647582
C	1.858912	0.148401	−1.128111
C	4.384771	−1.020630	0.810035
C	5.573111	−0.887661	1.761047
C	6.143957	0.527829	1.702534
C	6.478889	0.908005	0.261835
C	5.259837	0.710289	−0.638472
H	5.267139	−1.141085	2.778583
H	3.571446	−0.372723	1.158929
H	3.999902	−2.041948	0.803955
H	5.398757	1.229521	2.093108
H	7.026288	0.614173	2.339154
H	6.821196	1.943705	0.203483
H	7.296048	0.275846	−0.101604
H	4.474064	1.413987	−0.339804
H	5.505093	0.926274	−1.679562
H	6.346196	−1.605507	1.466842
N	4.689331	−0.631430	−0.564550
H	5.339162	−1.298763	−0.968258

TS

C	1.125632	2.316958	−0.271746
C	0.268632	3.184160	−0.186423
C	−0.967051	3.728823	−0.080735
C	−2.153755	3.987047	0.015682
C	−3.493993	3.797924	0.112417
C	−4.617666	3.337238	0.186828
C	0.037147	−3.177996	−0.320800
C	−1.050753	−3.714309	−0.252105
C	−2.387965	−3.938992	−0.157045
C	−3.589746	−3.785280	−0.063469
C	−4.816353	−3.209396	0.042407
C	−5.690296	−2.369091	0.127787
C	−6.320698	−1.167389	0.204330
C	1.061257	−2.284346	−0.376565
C	1.722539	−1.266266	−0.402180
C	−6.482661	0.036687	0.246071
C	1.917844	1.229187	−0.355886
C	−5.615779	2.417514	0.239585
C	−6.238825	1.373480	0.260700
C	2.183573	0.025754	−0.405608
C	4.734661	−0.988999	0.830855
C	6.243129	−1.070821	1.046679
C	6.878867	0.304453	0.855986
C	6.507922	0.880316	−0.508720
C	4.991885	0.893653	−0.693589
H	6.448534	−1.460883	2.045225
H	4.287374	−0.351150	1.600927
H	4.265497	−1.971134	0.903968
H	6.518651	0.977740	1.640793
H	7.962890	0.242484	0.961084
H	6.895953	1.894175	−0.621802
H	6.958357	0.270532	−1.298012
H	4.535651	1.590728	0.019446
H	4.714271	1.230365	−1.693220
H	6.670304	−1.776594	0.327384
N	4.382043	−0.405628	−0.461646
H	4.582934	−1.048720	−1.221007

DB

C	1.351097	2.152676	−0.107552
C	0.293801	2.790919	−0.043486
C	−0.989104	3.170370	0.019372
C	−2.181619	3.455573	0.076307
C	−3.521995	3.330264	0.123342
C	−4.711010	3.047312	0.159359
C	−0.063139	−2.447766	−0.232618
C	−1.129974	−3.027344	−0.214191
C	−2.431727	−3.415130	−0.177742
C	−3.645474	−3.455021	−0.131267
C	−4.957189	−3.106379	−0.065440
C	−5.931220	−2.381125	0.001291
C	−6.685705	−1.253183	0.074463
C	1.027450	−1.632574	−0.243011
C	1.907841	−0.798241	−0.243581
C	−6.831443	−0.045346	0.125970
C	2.566243	1.571565	−0.171148
C	−5.789770	2.231147	0.170750
C	−6.557319	1.283106	0.165441
C	2.836992	0.272815	−0.242039
C	4.693131	−0.626282	1.120690
C	6.091529	−1.213834	1.094992
C	7.089620	−0.231294	0.492411
C	6.621310	0.212964	−0.889514
C	5.220517	0.796632	−0.837335
H	6.363505	−1.476417	2.117387
H	4.636627	0.278026	1.727652
H	3.949530	−1.337416	1.476438
H	7.178271	0.642163	1.144163
H	8.076306	−0.688640	0.427723
H	7.282966	0.975508	−1.300327
H	6.643740	−0.632885	−1.583351
H	5.163826	1.674529	−0.196959
H	4.836805	1.065719	−1.818724
H	6.083668	−2.143713	0.518740
N	4.264797	−0.202012	−0.258627
H	4.294128	−1.033726	−0.855113

C_{22}–pip

vdW

C	-1.531369	2.535696	-0.907590
C	-0.736800	3.445822	-0.744834
C	0.392147	4.152286	-0.521779
C	1.543825	4.483040	-0.297685
C	2.869977	4.462572	-0.044454
C	4.015918	4.107413	0.171469
C	-0.777504	-3.353285	-0.816494
C	0.219923	-4.028915	-0.630430
C	1.488646	-4.424403	-0.390905
C	2.685605	-4.448516	-0.161617
C	3.963441	-4.089288	0.086091
C	4.982448	-3.451159	0.286861
C	5.866478	-2.446163	0.464774
C	5.123170	3.362199	0.375908
C	-1.644378	-2.329826	-0.974041
C	-2.133833	-1.216996	-1.060120
C	6.381399	-1.346607	0.573087
C	6.588887	-0.013416	0.624632
C	-2.115445	1.324447	-1.031170
C	5.898057	2.431574	0.515166
C	6.433891	1.195533	0.605971
C	-2.309155	0.121927	-1.081570
C	-5.727259	0.714538	-0.585216
C	-6.934035	0.926691	0.328288
C	-6.589158	0.538838	1.764616
C	-6.036041	-0.884055	1.814296
C	-4.859699	-1.030131	0.850365
H	-7.263180	1.966968	0.275757
H	-4.929114	1.407037	-0.293148
H	-5.980566	0.936407	-1.623147
H	-5.830840	1.229912	2.148848
H	-7.463601	0.635284	2.410611
H	-5.722705	-1.143461	2.828086
H	-6.821339	-1.591321	1.526887
H	-4.034930	-0.392843	1.191770
H	-4.487332	-2.056063	0.838774
H	-7.763274	0.306240	-0.027957
N	-5.174101	-0.634990	-0.520324
H	-5.839214	-1.292106	-0.915920

TS

C	-1.662607	2.305451	-0.286706
C	-0.849634	3.221399	-0.216685
C	0.322144	3.875567	-0.128146
C	1.474687	4.274601	-0.044838
C	2.819371	4.296979	0.043866
C	4.012872	4.051525	0.118029
C	-0.606609	-3.259719	-0.314352
C	0.438863	-3.883035	-0.258923
C	1.734872	-4.255790	-0.182204
C	2.950897	-4.283622	-0.104648
C	4.254925	-3.944282	-0.014351
C	5.297782	-3.317933	0.064142
C	6.205042	-2.320621	0.141432
C	5.181803	3.380764	0.182065
C	-1.580260	-2.322818	-0.360650
C	-2.226418	-1.291009	-0.384801
C	6.711323	-1.212804	0.195336
C	6.891459	0.124969	0.232174
C	-2.409740	1.197259	-0.354035
C	6.051167	2.525974	0.222547
C	6.663291	1.322857	0.239766
C	-2.692676	-0.008386	-0.393554
C	-5.467693	0.907587	-0.657897
C	-6.984647	0.896433	-0.482276
C	-7.365691	0.306150	0.873451
C	-6.735221	-1.073104	1.052718
C	-5.225308	-0.993477	0.847575
H	-7.368516	1.912776	-0.586088
H	-5.013022	1.593652	0.066457
H	-5.182260	1.254528	-1.651780
H	-7.008898	0.969481	1.668167
H	-8.450491	0.245907	0.970571
H	-6.947538	-1.473558	2.045606
H	-7.159850	-1.769772	0.323171
H	-4.780332	-0.364430	1.625948
H	-4.758906	-1.977492	0.912271
H	-7.431991	0.297389	-1.281399
N	-4.865113	-0.397490	-0.437503
H	-5.066360	-1.031528	-1.204614

DB

C	−1.902647	2.142915	−0.129266
C	−0.885141	2.856108	−0.075612
C	0.355547	3.328785	−0.022005
C	1.523426	3.721021	0.028119
C	2.859501	3.766257	0.074212
C	4.082426	3.671506	0.113800
C	−0.497771	−2.535882	−0.220002
C	0.548670	−3.158003	−0.202510
C	1.812352	−3.635469	−0.171807
C	3.015999	−3.821664	−0.134673
C	4.359911	−3.695106	−0.082825
C	5.482827	−3.223214	−0.028946
C	6.514720	−2.353439	0.032495
C	5.303852	3.112155	0.140230
C	−1.570616	−1.709463	−0.232325
C	−2.459896	−0.880259	−0.237046
C	7.079305	−1.272887	0.082304
C	7.302092	0.058092	0.126813
C	−3.059217	1.488138	−0.182462
C	6.296162	2.396050	0.155413
C	6.985280	1.237726	0.147617
C	−3.367760	0.196727	−0.240488
C	−5.736937	0.776688	−0.840275
C	−7.152537	0.229155	−0.882921
C	−7.630856	−0.181972	0.505861
C	−6.658304	−1.180908	1.123285
C	−5.244835	−0.630389	1.140007
H	−7.794430	1.002352	−1.304932
H	−5.657889	1.662205	−0.212577
H	−5.347289	1.020200	−1.826124
H	−7.697025	0.703277	1.144281
H	−8.628969	−0.614695	0.448193
H	−6.936592	−1.420683	2.149545
H	−6.674669	−2.119417	0.561362
H	−5.163616	0.281027	1.733595
H	−4.519521	−1.355461	1.505034
H	−7.196771	−0.626091	−1.564077
N	−4.808033	−0.238634	−0.246616
H	−4.860326	−1.078266	−0.829961

C_{18}–pip (gas-phase)

vdW

C	−0.768732	−3.297486	−0.030524
C	−1.943124	−3.638526	−0.012572
C	−3.284939	−3.543376	0.007058
C	−4.405119	−3.052646	0.023116
C	−5.375694	−2.121144	0.035765
C	−5.916682	−1.024222	0.041506
C	−6.060753	0.313309	0.040399
C	0.327522	−2.517782	−0.047821
C	1.017790	−1.508531	−0.059977
C	1.344998	−0.204018	−0.068232
C	1.216326	1.012768	−0.070672
C	0.601754	2.209726	−0.066316
C	−0.272908	3.064666	−0.055849
C	−1.505386	3.603796	−0.039187
C	−2.724615	3.698980	−0.020476
C	−4.017440	3.327501	0.000543
C	−5.011494	2.615095	0.018126
C	−5.763441	1.499686	0.032744
C	4.599872	−0.325316	−1.187038
C	6.074244	0.076923	−1.206071
C	6.410363	0.919017	0.023059
C	6.001131	0.187211	1.299649
C	4.529487	−0.219476	1.230420
H	7.473323	1.165628	0.043288
H	6.691759	−0.827777	−1.208361
H	6.301502	0.623353	−2.124218
H	3.980404	0.573965	−1.285886
H	4.359097	−0.973025	−2.031633
H	6.615388	−0.712027	1.417476
H	6.175639	0.812859	2.177914
H	4.238853	−0.791702	2.112963
H	3.907052	0.683038	1.214524
H	5.865205	1.867557	−0.034739
N	4.190874	−0.994955	0.042505
H	4.616708	−1.914108	0.095230

TS

C	-0.588443	-3.012393	-0.293269
C	-1.740320	-3.414432	-0.220409
C	-3.081496	-3.466675	-0.120632
C	-4.237133	-3.079420	-0.020152
C	-5.305232	-2.268210	0.088741
C	-5.901825	-1.203022	0.170433
C	-6.140614	0.119168	0.234552
C	0.489893	-2.206717	-0.347651
C	1.243334	-1.245542	-0.372817
C	1.760693	0.014292	-0.371260
C	1.479022	1.228723	-0.306017
C	0.603480	2.228991	-0.204685
C	-0.325050	3.032055	-0.104904
C	-1.602131	3.419196	0.005785
C	-2.820226	3.533335	0.102580
C	-4.091710	3.111876	0.184097
C	-5.115767	2.441035	0.238184
C	-5.831845	1.303835	0.252382
C	4.498625	0.926409	-0.696314
C	6.018699	0.868043	-0.568417
C	6.425513	0.255492	0.770031
C	5.758845	-1.105211	0.961748
C	4.246745	-0.978124	0.806001
H	7.510442	0.162123	0.831978
H	6.425458	0.264057	-1.385806
H	6.430625	1.872820	-0.677073
H	4.081027	1.618435	0.043859
H	4.188076	1.288869	-1.676947
H	6.140160	-1.810874	0.216572
H	5.991682	-1.518807	1.944593
H	3.747275	-1.945205	0.880321
H	3.843565	-0.339133	1.598659
H	6.115873	0.923461	1.580356
N	3.864697	-0.362036	-0.463412
H	4.014599	-0.997715	-1.240661

DB

C	-0.701290	-2.609248	-0.000025
C	-1.846182	-3.033786	-0.000026
C	-3.182108	-3.205114	-0.000018
C	-4.368109	-2.906736	-0.000007
C	-5.523256	-2.214826	0.000001
C	-6.126370	-1.147875	0.000011
C	-6.402744	0.167698	0.000019
C	0.426460	-1.867880	-0.000024
C	1.351895	-1.073199	-0.000025
C	2.225157	0.022146	-0.000020
C	1.942673	1.324454	-0.000006
C	0.790566	1.982762	0.000004
C	-0.246861	2.672260	0.000004
C	-1.529639	3.000396	0.000004
C	-2.747641	3.218001	0.000004
C	-4.038395	2.889220	0.000003
C	-5.168388	2.396056	0.000013
C	-5.954623	1.312382	0.000020
C	4.387437	0.102535	-1.239057
C	5.844189	-0.325505	-1.249547
C	6.570306	0.162047	0.000039
C	5.844159	-0.325622	1.249562
C	4.387407	0.102420	1.239076
H	7.602345	-0.187951	0.000036
H	5.907321	-1.416804	-1.314298
H	6.308360	0.069566	-2.153675
H	4.270578	1.186547	-1.230353
H	3.823936	-0.295965	-2.081462
H	5.907291	-1.416928	1.314216
H	6.308305	0.069365	2.153738
H	3.823884	-0.296156	2.081430
H	4.270549	1.186433	1.230469
H	6.599411	1.255377	0.000092
N	3.694137	-0.373378	-0.000022
H	3.711208	-1.395356	-0.000068

Full Gaussian 16 reference

Frisch, M. J.; Trucks, G. W.; Schlegel, H. B.; Scuseria, G. E.; Robb, M. A.; Cheeseman, J. R.; Scalmani, G.; Barone, V.; Petersson, G. A.; Nakatsuji, H.; Li, X.; Caricato, M.; Marenich, A. V.; Bloino, J.; Janesko, B. G.; Gomperts, R.; Mennucci, B.; Hratchian, H. P.; Ortiz, J. V.; Izmaylov, A. F.; Sonnenberg, J. L.; Williams-Young, D.; Ding, F.; Lipparini, F.; Egidi, F.; Goings, J.; Peng, B.; Petrone, A.; Henderson, T.; Ranasinghe, D.; Zakrzewski, V. G.; Gao, J.; Rega, N.; Zheng, G.; Liang, W.; Hada, M.; Ehara, M.; Toyota, K.; Fukuda, R.; Hasegawa, J.; Ishida, M.; Nakajima, T.; Honda, Y.; Kitao, O.; Nakai, H.; Vreven, T.; Throssell, K.; Montgomery, J. A., Jr.; Peralta, J. E.; Ogliaro, F.; Bearpark, M. J.; Heyd, J. J.; Brothers, E. N.; Kudin, K. N.; Staroverov, V. N.; Keith, T. A.; Kobayashi, R.; Normand, J.; Raghavachari, K.; Rendell, A. P.; Burant, J. C.; Iyengar, S. S.; Tomasi, J.; Cossi, M.; Millam, J. M.; Klene, M.; Adamo, C.; Cammi, R.; Ochterski, J. W.; Martin, R. L.; Morokuma, K.; Farkas, O.; Foresman, J. B.; Fox, D. J. *Gaussian 16, Rev. C.01*; Gaussian, Inc., Wallingford, CT, **2019**. http://www.gaussian.com

Full POLYRATE reference

Zheng, J.; Bao, J. L.; Meana-Pañeda, R.; Zhang, S.; Lynch, B. J.; Corchado, J. C.; Chuang, Y.-Y.; Fast, P. L.; Hu, W.-P.; Liu, Y.-P.; Lynch, G. C.; Nguyen, K. A.; Jackels, C. F.; Fernandez Ramos, A.;Ellingson, B. A.; Melissas, V. S.; Villà, J. Rossi, I.; Coitiño, E. L.; Pu, J.; Albu, T. V.; Ratkiewicz, A.; Steckler, R.; Garrett, B. C.; Isaacson, A. D.; Truhlar, D. G. *Polyrate*-version 2017-C; University of Minnesota: Minneapolis, 2017. https://comp.chem.umn.edu/polyrate/
